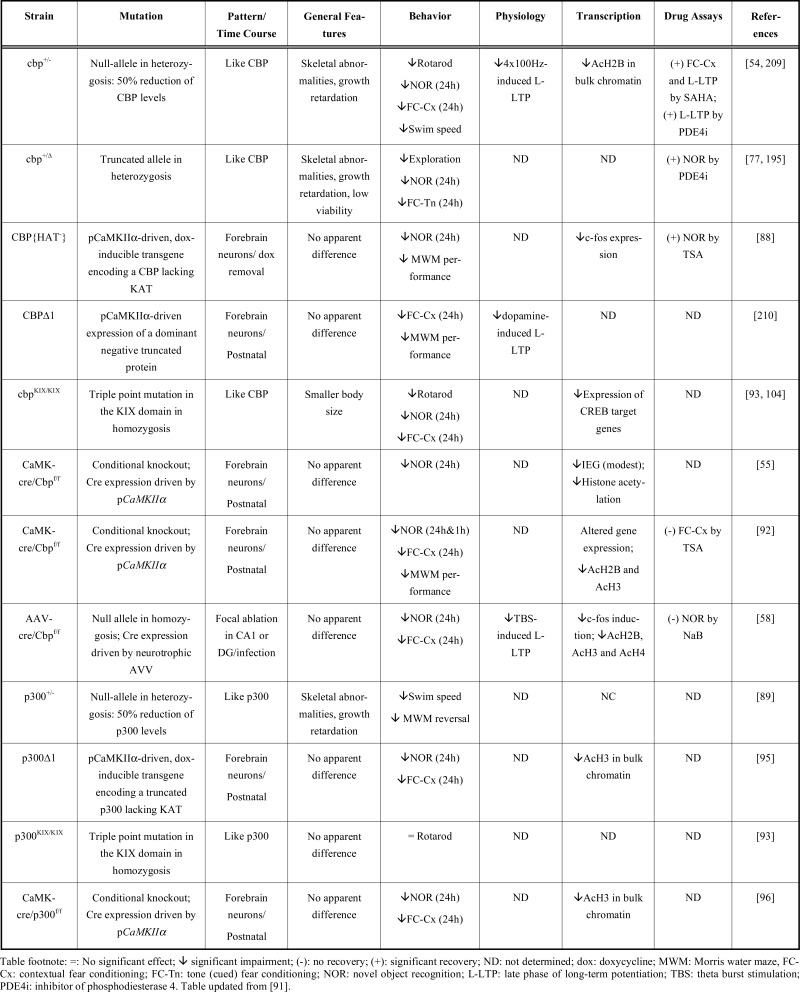# Lysine Acetyltransferases CBP and p300 as Therapeutic Targets in Cognitive and Neurodegenerative Disorders

**DOI:** 10.2174/13816128113199990382

**Published:** 2013-08

**Authors:** Luis M Valor, Jose Viosca, Jose P. Lopez-Atalaya, Angel Barco

**Affiliations:** Instituto de Neurociencias (Universidad Miguel Hernández-Consejo Superior de Investigaciones Científicas). Av. Santiago Ramón y Cajal s/n. Sant Joan d’Alacant. 03550. Alicante, Spain

**Keywords:** Histone acetylation, CBP, p300, Rubinstein-Taybi syndrome, Huntington’s disease, Alzheimer’s disease, HDACi, KATe, KATi.

## Abstract

Neuropsychiatric pathologies, including neurodegenerative diseases and neurodevelopmental syndromes, are frequently associated with dysregulation of various essential cellular mechanisms, such as transcription, mitochondrial respiration and protein degradation. In these complex scenarios, it is difficult to pinpoint the specific molecular dysfunction that initiated the pathology or that led to the fatal cascade of events that ends with the death of the neuron. Among the possible original factors, epigenetic dysregulation has attracted special attention. This review focuses on two highly related epigenetic factors that are directly involved in a number of neurological disorders, the lysine acetyltransferases CREB-binding protein (CBP) and E1A-associated protein p300 (p300). We first comment on the role of chromatin acetylation and the enzymes that control it, particularly CBP and p300, in neuronal plasticity and cognition. Next, we describe the involvement of these proteins in intellectual disability and in different neurodegenerative diseases. Finally, we discuss the potential of ameliorative strategies targeting CBP/p300 for the treatment of these disorders.

## 1 CHROMATIN ACETYLATION AND COGNITION

### 1.1 Epigenetic Regulation of Cognitive Function

Epigenetic mechanisms, such as DNA methylation and post-translational modification of histones, provide a molecular interface between genes and environment that allows the modulation of gene expression by experience. These mechanisms are known to play a key role in the specification of cell lineages during embryonic development. Throughout cell differentiation, cells with the same genome acquire unique properties by switching on and off specific gene programs; their acquired gene expression profiles will then be inherited by daughter cells along successive rounds of division [1]. It has been known for years that this process depends on the epigenetic modification of the chromatin of differentiating cells. In comparison, the idea that epigenetic mechanisms are involved in cognition is relatively new. The relevance of these mechanisms to brain function was long overlooked by neuroscientists because adult neurons do not divide and are thought to be terminally differentiated. However, studies during the last decade have indicated that these mechanisms might allow neurons to customize their gene expression patterns according to experience, widening the computational and storage capabilities of neural networks. Epigenetic mechanisms also represent a suitable basis for the phenotypic variation in behavioral and cognitive traits observed in humans. Thus, in monozygotic twins who share the same genomic sequence, the differences in the contents and distributions of epigenetic marks grow throughout life along with the discordance of psychological traits [2]. 

Among these epigenetic mechanisms, the acetylation and deacetylation of histone tails play a prominent and particularly well-studied role. The N-terminus of the four nucleosome histones (H2A, H2B, H3 and H4) is unstructured and has high intrinsic flexibility that enables the dynamic interaction of histones with DNA. The addition of an acetyl group to lysine residues located in these tails neutralizes its basic charge and loosens up the contacts between histones and DNA, which causes the relaxation of the chromatin and may facilitate the recruitment of transcription factors and the basal transcriptional machinery to specific DNA sequences. Thus, the degree of chromatin compaction is thought to contribute to both the definition of global patterns of transcriptional competence and to the local regulation of transcription initiation and elongation at specific loci [3]. Histone acetylation is mediated by histone acetyltransferase activities (HATs) and has been generally associated with gene activation, whereas histone deacetylation is mediated by histone deacetylases (HDACs) and has been associated with gene repression. However, this association is more complex than initially expected because both HATs and HDACs bind to gene promoters regardless of their transcriptional activity [4]. The term *poised* (also referred to as *primed* or *bookmarked*) gene has recently been coined to explain the presence of histone modifications associated with transcriptionally active genes at promoters of inactive genes that likely enable their induction in response to particular stimuli [4-6]. 

Importantly, the balance between histone acetylation and deacetylation is very dynamic. In neurons, chromatin acetylation is modulated in response to neuronal activity and during plasticity processes [7]. Extensive correlative evidence supports a role for histone acetylation in memory storage that seems highly conserved through evolution [8-13]. Although we present below some seminal findings supporting a role for histone acetylation in plasticity and memory, we do not intend to review the extensive literature on this topic here because several excellent review articles have recently been published [14-18].

Both in invertebrates and vertebrates, memory acquisition has been associated with changes in histone acetylation in the neuronal nucleus. In some of these studies, the changes were restricted to specific gene promoters and investigated through chromatin immunoprecipitation (ChIP) assays, in others the changes were so general that they could be detected at the bulk chromatin level by western blot or immunohistochemistry. For example, in *Aplysia californica,* stimuli that trigger long-term facilitation (LTF) or long-term depression (LTD), two forms of plasticity that are thought to be cellular correlates of memory formation in this organism, bidirectionally regulate the acetylation of H3-lysine (K)14 and H4-K8 in the promoter of the gene encoding the CCAAT-box-enhanced binding protein (C/EBP), a transcription factor involved in the maintenance of LTF [19]. More recently, experiments in another invertebrate, the crab *Chasmagnathus*, demonstrated that strong context-signal training triggers increases in bulk histone H3 acetylation in the neurons involved in this form of learning [20]. Experiments in rodents indicate that several nucleosome positions are acetylated during learning tasks. These marks are associated with specific memory stages and have been detected in the chromatin of neuronal types known to be engaged in that type of memory. For instance, bulk H3 acetylation increases in the hippocampus shortly after contextual fear conditioning [21], whereas spatial memory acquisition in a water maze task is associated with acetylation of hippocampal H2B and H4 [22]. H3 acetylation increases have also been observed in the lateral amygdala after cued fear conditioning training [23], and a variety of modifications have been recently reported to occur during object recognition memory formation and recall [24]. Like in invertebrates, some studies in rodents have demonstrated that the changes in acetylation can be restricted to the promoters of genes known to be involved in memory formation, such as that encoding the neurotrophin BDNF. Thus, histone H3 was hyperacetylated at the BDNF gene promoter after retrieval of contextual fear memory, and this hyperacetylation was required for reconsolidation of that memory [25], whereas extinction of tone fear memory triggered H4 hyperacetylation at the same promoter in neurons of the prefrontal cortex [26]. 

Collectively, these data indicate that an array of histone positions can be differentially acetylated during memory formation. However, we should note that all the histone acetylation marks that have been reported to occur during memory formation thus far are transient. These marks may be part of the regulatory mechanisms that control the timing of activity dependent events, but it is unlikely that they directly participate in encoding the memory [9]. More stable epigenetic marks, such as DNA methylation and other posttranslational histone modifications (methylation, ubiquitination, sumoylation or more stable acetylation events) might correlate better with the duration of the memory process [27]. 

### 1.2 Histone Acetyltransferases and Deacetylases as Memory Gates

As previously mentioned, histone acetylation is regulated by the opposing enzymatic activities of HATs and HDACs. However, despite the names of these enzymes, histones are not their only substrates. Non-histone substrates include other nuclear proteins like transcription and chromatin remodeling factors and diverse cytoplasmic and mitochondrial proteins. In fact, computational predictions [28] and high throughput mass spectrometry analyses [29] indicate that hundreds of proteins may be susceptible to acetylation. For this reason, it has recently been proposed that the term HAT should be replaced by KAT, wherein the K stands for lysine [30]. We will use this terminology in the following sections. Similarly, HDAC could have been renamed as KDAC, but the scientists proposing the new nomenclature felt that the HDAC families already had a coherent nomenclature and that renaming them would serve to confuse rather than clarify [30]. 

**Fig. (1):**
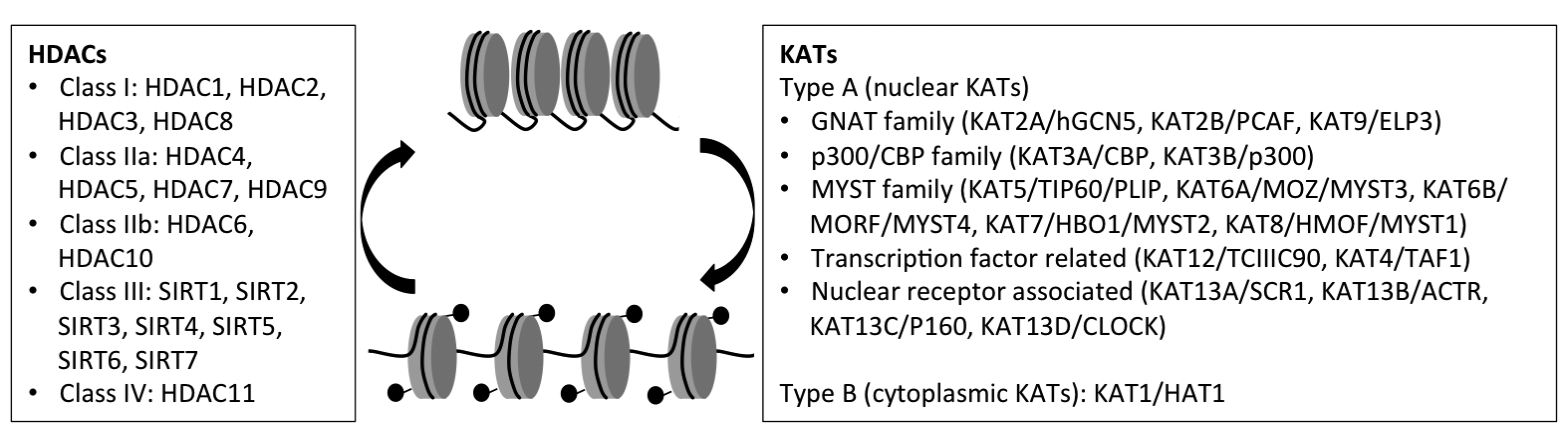
**KATs and HDAC classes.** Schematic depicting the balance of KAT and HDAC activities and the impact of this balance on chromatin conformation. The presence of acetyl groups (black balls) in the nucleosome histone tails relaxes the chromatin and facilitates the access of transcription factors. The boxes show the general classification of KAT and HDAC proteins and list representative examples for each group. For the KATs, we used the new nomenclature, but the former name or names are also indicated [30]. See the review articles [31, 156] for additional details.

KAT proteins catalyze the formation of an amide bond between the ε-amino group of a lysine residue and acetyl-Coenzyme A. They have been grouped into two classes (Fig. **1**). Type A KATs are thought to acetylate nucleosomal histones during or in close temporal proximity to transcriptional events, whereas type B KATs primarily acetylate newly synthesized histones in the cytoplasm before their assembly into chromatin. Type A KATs are separated into five families based on similarities in their primary sequences [31, 32]. Counteracting KAT activity, HDAC proteins remove acetyl groups from acetyl-lysines and are classified, according to protein function and sequence similarities, into five families that includes HDAC1 to 11 and sirtuins SIRT1 to 7 [33] (Fig. **1**).

Both KATs and HDACs have been postulated to be key regulators of memory storage. Although most KATs and HDACs are ubiquitously expressed, some are expressed at particularly high levels in brain areas involved in learning and memory, such as the hippocampus and prefrontal cortex [34]. Deficiencies in KAT activity have been associated with intellectual disability (ID) and cognitive impairment; thus, KATs are thought to be positive regulators of cognitive processes. This is the case for the KAT3 proteins CBP and p300, which will be discussed in detail in the next section, and for KAT2B (a.k.a. p300/CBP-associated factor, PCAF) [35, 36]. Based on these studies, PCAF and CBP/p300 seem to have non-overlapping functions in memory formation. Conversely, different HDAC activities have been postulated to be negative regulators of memory formation. The evidence is especially robust for Class I HDACs. Thus, HDAC2 levels seem to regulate memory formation in a bidirectional manner because contextual and cued fear memory are enhanced in HDAC2 knockout mice and impaired in mice overexpressing HDAC2 [37]. Moreover, HDAC2 knockout mice show increased numbers of synapses and facilitated L-LTP, while mice overexpressing HDAC2 show the opposed phenotype [37]. Similar to HDAC2, conditional deletion of HDAC3 in the CA1 region, achieved by injecting adeno-associated virus expressing cre recombinase in HDAC3 floxed mice, leads to a persistent enhancement of LTM [38]. Strikingly, two recent studies have shown that the loss of either HDAC4 [39] or HDAC5 [40] impairs memory function, suggesting that Class IIa enzymes, in contrast to Class I enzymes, positively regulate memory consolidation. The family of NAD^+^-dependent Class III HDACs includes sirtuin proteins that have also been postulated to be positive regulators of memory formation. Thus, mice bearing a conditional brain deletion of *Sirt1* show deficits in contextual and cued fear memory [41].

### 1.3 KAT3 Proteins: Molecular Structure, Function and Regulation

CBP and p300 are the only members of the CBP/p300 or KAT3 family [30]. Both are large ubiquitously expressed nuclear proteins with an approximate molecular mass of 250 kDa. CBP was named after its initial description as an interacting partner of the transcription factor CREB (cAMP responsive element binding) [42], whereas p300 was initially described as the host factor interacting with the protein E1A from adenovirus type 5 [43, 44]. It was later found that both proteins really interact with hundreds of proteins with different functions [32, 45-47]; thus, it has recently been proposed that CBP and p300 should be renamed KAT3A and KAT3B, respectively, in an attempt to standardize the nomenclature of chromatin-remodeling enzymes [30]. 

**Fig. (2):**
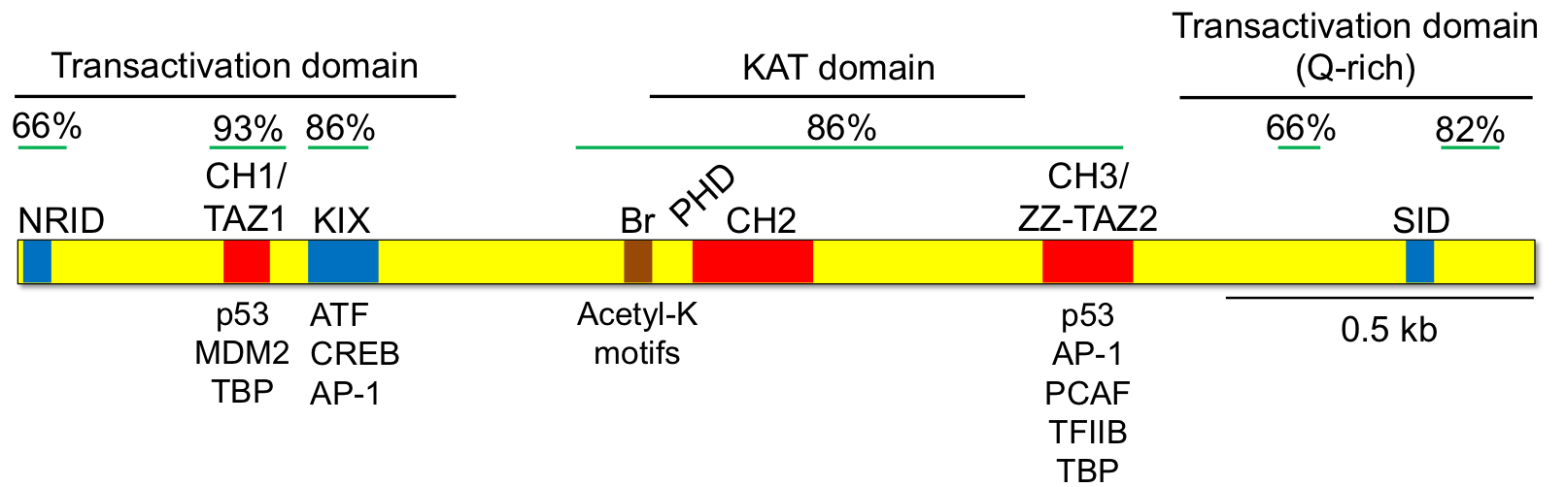
**Structure of KAT3 proteins.** CBP and p300 share a number of structural domains including three cysteine/histidine rich regions (CH1-CH3) for protein-protein interaction, the KIX domain that mediates the interaction with CREB and other transcription factors, and the KAT domain. The domains of highest homology and the percentage of amino acid identity between the two proteins are indicated. Regions of high homology between the human CBP and p300 proteins expressed as % identity. NRID, nuclear hormone receptor interacting domain; CH1-3, cysteine/histidine-rich regions 1-3; TAZ1-2, transcriptional adaptor Zn-finger domain 1-2; KIX, kinase inducible domain; Br, bromodomain; PHD, plant homeodomain; ZZ, ZZ-type Zn-finger domain; SID, SRC- 1 interacting domain; MDM2, p53 E3-ubiquitin protein ligase homolog; ATF, activation transcription factors; TBP, TATA-binding protein. Figure modified from [52].

KAT3 proteins have diverse functions related to transcription activation and regulation. Thus, they are usually described as molecular scaffolds that bring different proteins together to the promoters. Their large size (over 2400 aas) and modular organization enable interaction with several proteins at the same time. For example, the interaction of CBP with MAPKs and the E-Cdk2 complex not only promotes the phosphorylation of CBP but also the phosphorylation of several CBP-interacting transcription factors [48, 49]. The following domains can be distinguished in both CBP and p300 (Fig. **2**): (i) three cysteine/histidine-rich regions (CH1 to CH3) that bind zinc and are involved in protein-protein interactions; (ii) a lysine acetyltransferase (KAT) domain in the center of the protein; (iii) a bromodomain (BD) that binds acetylated lysines in histones and specific transcription factors [50]; (iv) two transactivation domains located at either end of the protein; and (v) multiple specific interaction domains for different transcription factors, such as the KIX domain that mediates the interaction between CBP/p300 and CREB phosphorylated at Ser133 [51]. An important consequence of this structure is that CBP/p300 can act as a molecular bridge between DNA-binding transcription factors and components of the basal transcription machinery, such as the TATA-box-binding protein (TBP) and the RNApol II complex. In addition, the KAT activity of CBP/p300 can relax the configuration of the chromatin around the bound DNA sequences by acetylation of histones. These are thought to be the molecular mechanisms responsible for the function of KAT3 proteins as transcriptional co-activators.

Because CBP and p300 are highly homologous proteins (with >70% of overall identity) and share most of their known functional domains [52], it is reasonable to assume that both proteins have highly overlapping functions through their interactions with similar partners and substrates. However, these two proteins also play unique roles during development, hematopoiesis, the response to DNA damage, and other processes [46, 53]. Future experiments should determine which protein interactions effectively occur in the nervous system *in vivo* and distinguish CBP from p300 and both proteins from other KAT activities. 

The substrate specificity and preferences of KAT3 proteins also remain unclear. Experiments in CBP-deficient mice [54, 55] and human cell lines [56] with reduced CBP function indicate that the N-terminal tail of histones H2A and H2B, especially the latter, are more sensitive to changes in CBP activity than those of histone H3 and H4. Previous biochemical assays and studies in cultured cells have also identified histone H2B as one substrate for the KAT activity of CBP [3, 57]. In particular, a comprehensive analysis carried out by MacManus and Henzdel in transfected cells indicated that CBP and p300 have both common and specific targets. As such, overexpression of CBP results in increased levels of AcH4K12 but not AcH4K8 while p300 overexpression promotes hyperacetylation of histone H4K8 but not H4K12 [57]. In contrast, experiments in which CBP was specifically knocked-down in the CA1 subfield of the mouse hippocampus produced reduced levels of AcH2BK12, AcH3K14 and AcH4K8 but normal levels of AcH4K12 [58]. Overall, the following histone residues have been identified as substrates of CBP and/or p300 in different studies: H2AK5, H2B(K5, K12, K15, K20), H3(K14, K18, K27) and H4(K5, K8) [55, 58-60]. In addition to histones, other nuclear proteins are also acetylated by KAT3 proteins, such as the tumor suppressors p53 [61, 62] and pRb [63], components of the RNApol II complex (TFIIE and TFIIF) [64] and diverse transcription factors [65-67].

Another important issue that needs further clarification is how the activity of KAT3 proteins is regulated by neuronal activity. These proteins are found at the convergence of various important signaling cascades and are targets of different posttranslational modifications that modulate their activity. Thus, the transactivation potential of CBP is increased by PKA, CaMKIV and p42/44 MAPKs, while the ability to recruit specific transcription factors is selectively increased after PKC phosphorylation [68]. Furthermore, CBP is phosphorylated at Ser301 by CaM kinases [69] and is methylated at Arg residues by the methylase CARM1 [70]. While CBP phosphorylation contributes to CBP-dependent transcription, its methylation appears to block its interaction with CREB and prevent CREB-dependent gene expression. The significance of these regulatory steps *in vivo* is, however, unclear. Another regulatory mechanism of KAT3-dependent transcription relies on the limited supply of these proteins within the cell. As a consequence, different transcription factors compete for binding to specific sites. For example, because of this competition, CREB activity can be indirectly inhibited by viral proteins that are not functionally or structurally related to CREB, but that sequester CBP from its molecular partners [71]. DNA context can also regulate KAT3 activity. Because CBP and p300 do not bind directly to DNA, their recruitment to gene promoters is highly dependent on transcription factor activities. Thus, a recent study in CBP/p300 depleted cells revealed an inverse correlation between CREB occupancy (determined by the number of CREB binding sites at specific promoters) and CBP/p300 dependence for gene induction in response to cAMP [72]; this result indicates that these proteins likely compete with other cofactors to regulate CREB-dependent expression. Intriguingly, the alternative CREB cofactors do not have KAT activity, which puts the relevance of increased histone acetylation for gene induction into question [73]. 

## 2 KAT3 DYSREGULATION IN NEUROLOGICAL DISORDERS

**Fig. (3):**
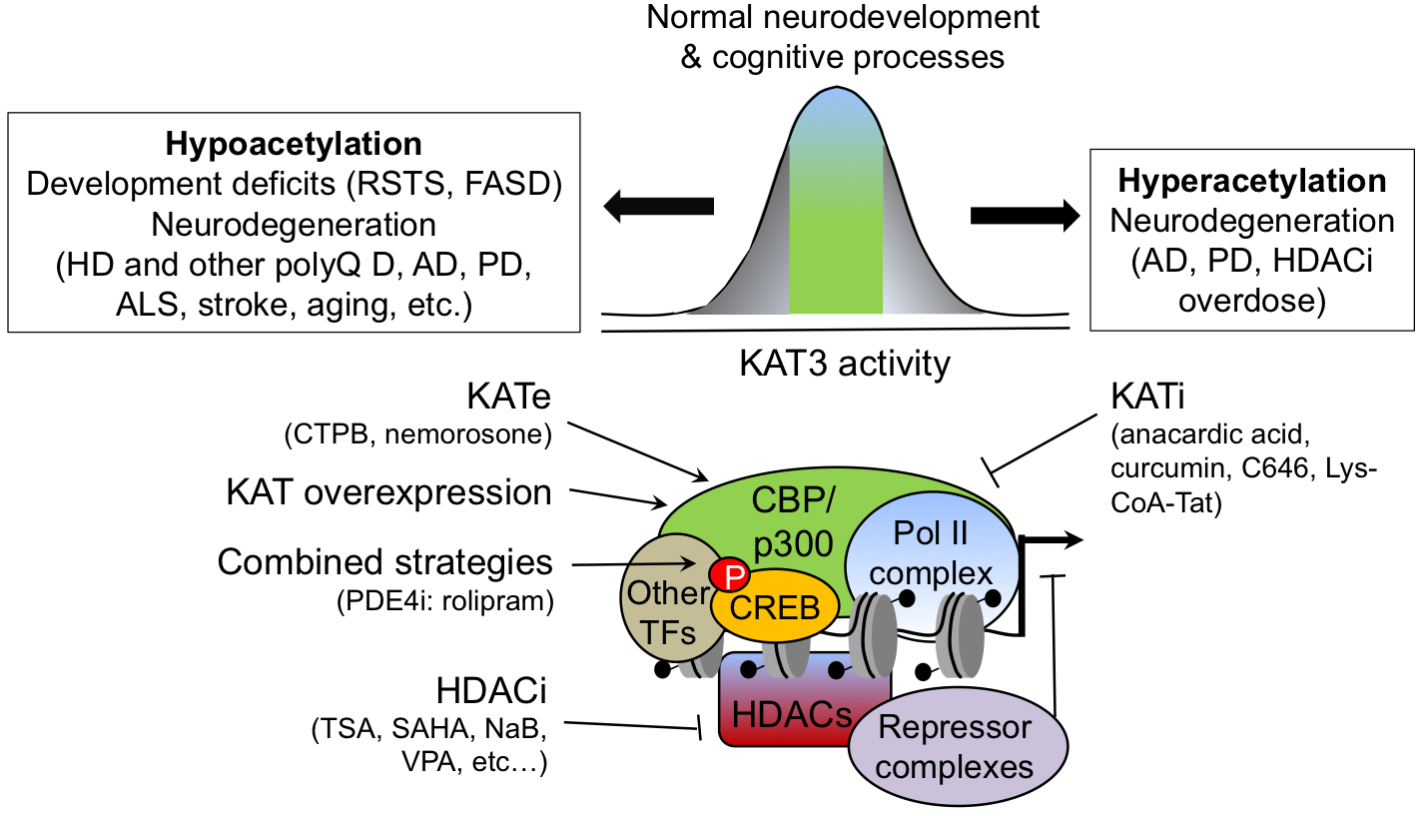
**Summary of neuropathologies related to KAT3 proteins and therapeutic approaches targeted to these proteins.** The balance between KATs and HDACs define the optimal level of chromatin acetylation required for developmental and cognitive functions. The alteration of this balance, favoring either histone hyperacetylation (right box) or hypoacetylation (left box), may result in cognitive impairment and/or deleterious effects. This is the case of well-known pathological conditions like the Rubinstein-Taybi syndrome (RSTS), Fetal Alcohol Spectrum Disorder (FASD), Huntington’s disease (HD), Alzheimer’s disease (AD), Parkinson’s disease (PD), amyotrophic lateral sclerosis (ALS) and other neurological disorders. Therapeutic approaches aimed at restoring the balance, including HDAC inhibitors (HDACis), KAT enhancers (KATes) and inhibitors (KATis) and genetic overexpression of KAT3 genes, are indicated under the corresponding boxes.

Given the multiple interactions of KAT3 proteins and the role of downstream genetic cascades in neuroprotection, including CREB-dependent gene expression [74, 75], it is not surprising that altered KAT activity had been linked to diverse neurological disorders (Fig. **3**). In this section, we will focus exclusively on the consequences of dysregulation of KAT3 proteins in brain function. Their role in other pathologies, like cancer, has been reviewed elsewhere [76].

### 2.1 Neurodevelopmental Disorders

During development, CBP and p300 control the proliferation and differentiation of different cell lineages, including those within the nervous system. In mammals, CBP and p300 are first required for neural tube closure at the three-layer embryonic stage. Homozygous mice bearing *cbp* or *p300 *null alleles show exencephalia and die early during development between E9 and E12.5 [77-79]. At that time, the neural plate is folding to generate the neural tube and highly expresses both proteins [80, 81]. Strikingly, the double heterozygous mutation for CBP and p300 is also lethal [79], indicating that these two proteins play redundant roles during early development and can be interchangeable. Afterwards, their expression levels decay [80, 81], but they are still involved in the differentiation of a variety of cellular types, such as motoneurons, cortical progenitors and astrocytes [82]. Given this context, it is not surprising that the deficiency of these proteins is associated with neurodevelopmental disorders.

#### 2.1.1 The Rubinstein-Taybi Syndrome

Rubinstein-Taybi syndrome (RSTS) is a complex autosomal dominant disease affecting 1 out of 100,000 newborns that is characterized by ID among other medical conditions [83, 84]. Most RSTS cases are associated with mutations in the genes encoding CBP (*CREBBP*, 60% of cases) [85] and p300 (*EP300*, 3% of cases) [86]. RSTS patients have a characteristic facial appearance and other skeletal abnormalities, such as defects in angulation, positioning or duplication of phalanges and thumb halluces that produce the characteristic broad thumbs and big toes that are used in diagnosis. Neuroanatomical defects, including agenesis of the corpus callosum, Dandy-Walker malformation, cortical clefts and subtler anatomical defects, have sometimes been observed in the brains of RSTS patients. Psychological studies have shown that RSTS patients have low levels of intelligence, short attention spans and poor motor coordination [87]. Although developmental alterations clearly underlie certain clinical manifestations of RSTS such as skeletal defects and growth retardation, experiments in animal models indicate that the lack of KAT3 proteins in the adult brain also contributes to the cognitive impairment in RSTS [88]. 

Mice bearing genetic disruptions similar to those found in RSTS patients express phenotypes reminiscent of some clinical manifestations of this syndrome. Therefore these mice represent valuable experimental models for the investigation of the molecular etiology of the syndrome and evaluation of therapeutic approaches. As indicated above, studies in mouse mutants have shown that CBP and p300 play roles both during embryonic development and in the adult brain, suggesting that the deficiency at both stages is likely to contribute to ID in humans. CBP and p300 hemizygous mice show some skeletal malformations that resemble those described in RSTS patients, but they have overall normal brain anatomy [54, 77, 89, 90]. The phenotype is more apparent in CBP deficient mice, which show more severe facial dysmorphia and alterations in phalanges and the trunk skeleton including scoliosis and asymmetry, and deficiencies or excesses of ribs and vertebras. These mutants also have reduced body weight and size, consistent with the impaired growth that characterizes RSTS. 

In attempts to model the cognitive deficits, the different mouse models of RSTS, including hemizygous mice, conditional knockout (cKO), transgenic mice expressing dominant negative variants and mice with focal depletion of the protein achieved through viral transduction (Table **1**), have been examined in a variety of learning and memory tasks. These experiments have revealed memory defects in the step-through passive avoidance, fear-conditioning, object recognition memory and Morris water maze tasks as well as other ID-related phenotypes [91]. Particularly remarkable are the experiments in transgenic mice with regulatable expression of a dominant negative CBP variant with a mutated KAT domain (referred to as CBP{HAT}) suggesting that intact KAT activity in the adult forebrain is required for the consolidation of some forms of explicit memory [88]. Recent experiments in different strains of cKO mice also support this view [55, 58, 92], although the reported memory deficits were in some cases weaker than those observed in hemizygous animals. Most CBP mutant strains showed specific deficits in long-term memory (LTM) but not in short-term memory (STM), suggesting a role for CBP in memory consolidation. However, a recent study on cKO mice in which CBP was completely inactivated in excitatory neurons of the postnatal forebrain revealed deficits in both LTM and STM [92], suggesting that the role of CBP in memory storage may also encompass memory encoding. This effect was however not observed in other similar CBP cKO mice [55, 58]. In addition to memory deficits, several CBP deficient strains, including *cbp*^+/- ^mice and *cbp*^kix/kix ^mice bearing a point mutation within the KIX domain that blocked CBP-CREB interaction, exhibited impaired motor skill learning [54, 93], that may resemble the difficulty in planning motor acts and executing locomotor skills that RSTS patients are reported to experience [94]. 

In comparison, the analysis of p300 deficient mice revealed weaker defects. Mice expressing a truncated *p300* protein (*p300*^∆1^) in the hippocampus, amygdala, cortex and cerebellum exhibited memory deficits in contextual fear conditioning and object recognition tasks [95]. Similar memory deficits have been also reported in cKO mice with a *p300* deletion restricted to CA1 and the cortex [96]. However, the analyses of *p300*^+/- ^mice bearing a null allele, a genetic condition closer to RSTS, revealed only mild impairments in the water maze and no impairment in fear conditioning or object recognition memory [89]. *P300*^+/-^ mice also exhibit mild motor deficits such as abnormal gait and reduced swimming speed. These observations are in agreement with the weaker ID in patients bearing mutations at *EP300* compared to *CREBBP* [97-99] and with the different prevalences of mutations in each of these genes among RSTS patients. In fact, genetic studies indicate that not all *EP300* mutations necessarily produce a RSTS condition [99]. 

Research on RSTS mouse models indicates that impaired neuronal histone acetylation is a critical hallmark of the disease that is likely to play a role in the etiology of the symptoms [100]. Notably, experiments in lymphoblastoid cell lines from RSTS patients have revealed similar histone acetylation deficits [56]. In addition, altered CREB-dependent transcription [101, 102] may also contribute to the cognitive deficits observed in RSTS. Supporting this view, CRE-dependent transcription is impaired in blood cell lines from RSTS patients bearing mutations in the KAT domain of CBP [103] and in neurons of transgenic mice expressing a dominant negative CBP variant [88, 104]. Furthermore, enhancement of CREB activity ameliorates synaptic plasticity deficits in *cbp*^+/-^ mice [54]. However, it should be noted that experiments in CBP heterozygous and forebrain-specific cKO mice indicate that the reduction or loss of CBP affects differently different activity-driven gene expression programs related to CREB. Thus, whereas the hippocampal induction of immediate early genes in response to novelty is only moderately affected by CBP deficiency [55], the transcriptional program induced in response to environmental enrichment (EE) is clearly impaired [90]. 

The requirement for p300 in CREB-dependent transcription is also not clear. Although p300 can activate CRE dependent transcription [105], experiments in fibroblasts from *p300*^-/-^ null mice stimulated with the catalytic subunit of PKA have revealed normal activation of a CRE-luciferase reporter [79]. Gene expression profiling in the hippocampus of p300 heterozygous mice also did not reveal a specific impairment of CREB-dependent transcription [89]. 

#### 2.1.2 Fetal Alcohol Spectrum Disorder

A second neurodevelopmental disorder, in this case with an environmental origin, has been recently associated with deficits in CBP. Exposure to alcohol during pregnancy causes alterations in the development of the cerebellum that lead to Fetal Alcohol Spectrum Disorder (FASD), which is characterized by deficits in motor coordination, balance and cerebellar-dependent learning tasks [106]. Experiments in rats indicate that CBP protein and histone H3 and H4 acetylation levels are all reduced in the cerebellum of ethanol-treated pups [107]. The involvement of KAT activity in this disorder deserves further examination, including assessment of the efficacy of drugs that increase histone acetylation (see Section 3) in animal models of this disease.

### 2.2 Neurodegenerative Diseases

Several neurodegenerative conditions have been associated with a reduction in both CBP activity and histone acetylation levels. Moreover, the restoration of acetylation levels, mainly by pharmacological means, as we will discuss in Section 3, concurs with the reversal of neuropathological traits. Both lines of evidence have led to the hypothesis that unbalanced KAT/HDAC activities contribute to the etiology of neurodegenerative disorders [108]. On the other hand, conditional CBP knockout mice exhibit a dramatic reduction in neuronal histone acetylation, but this does not seem to affect neuronal viability, indicating that sustained low levels of histone acetylation do not necessarily cause neuronal death *per se* [55, 92]. This conclusion is in agreement with the lack of brain atrophy in RSTS patients and models. Importantly, the primary cause of the lethality observed during development of KAT3 homozygous knockout mice is stalled proliferation. Because KAT3 proteins are required for DNA replication checkpoints, their depletion may trigger the death of proliferative cells [109, 110] or at least the loss of their proliferation capability [72] as a consequence of a mitotic failure. The mechanisms by which the loss of CBP and p300 could hamper the viability or survival of postmitotic neurons is less clear. The requirement of normal levels of KAT3 activity for adult neuron survival is, therefore, still under debate. Regardless of this requirement, neuronal malfunction could contribute to the mood changes, memory impairment and other neurological traits observed in patients with neurological conditions associated with KAT3 deficiency. 

#### 2.2.1 Huntington’s Disease 

Huntington’s disease (HD) is an inheritable fatal neurodegenerative disorder that is caused by expansions of CAG repeats in the Huntingtin gene (*HTT*) that lead to an aberrant enlargement of a poly-Q track at the protein level [111]. Experiments in animal and cellular models have associated neuropathology in HD with the loss of CBP function and impaired neuronal histone acetylation [112-117]. Thus, it has been proposed that CBP is sequestered in the protein aggregates of mutant Htt (mHtt) observed in the brain tissue of patients and in most experimental models of the disease. This sequestration would cause the depletion of CBP from the cell nucleus resulting in transcriptional dysregulation and cellular toxicity. However, this effect has not been reproduced in all experimental models of HD [118-121]. In addition to the proposed sequestration in aggregates, soluble mHtt can promote the degradation of CBP [122]. In fact, a recent comparison of aggregated and soluble mHtt suggests that the soluble form is more efficient than the aggregates in reducing CBP levels [123]. Interestingly, the turnover of p300 was not affected by mHtt [122]. A third mechanism by which mHtt can interfere with CBP function is the direct inhibition of its KAT activity [124, 125]. 

Regardless of the specific molecular mechanism underlying the disruption of CBP function in HD, different processes downstream of CBP can contribute to the cognitive deficits observed in patients and mouse models of the disease. First, the reduction in neuronal histone acetylation levels could interfere with cognitive processes, as discussed in section 1.1. Second, the disruption of CREB-dependent gene expression could affect memory consolidation. Interestingly, a number of studies have confirmed that CREB-dependent transcription is altered in HD, but with important divergences depending on the model; CREB-dependent transcription has been shown to be inhibited in some studies [126-129] and enhanced in others [119, 130]. Third, the interference with CBP-dependent acetylation of non-histone substrates, such as p53, which is also involved in HD [114, 131], and Htt itself, could accelerate the progression of the pathology. Because the turnover of Htt depends on the proper balance of CBP and HDAC1 activities, the reduction of CBP could cause an accumulation of mHtt [132]. Fourth, the instability of CAG repeats during germline transmission, a process that depends on HDAC and KAT3 proteins [133, 134], would produce longer poly-Q stretches and could contribute to explaining the phenomenon of anticipation (i.e., worsening of the disease in the next generation as reflected by earlier onset). 

In agreement with the role for CBP in HD outlined above, the overexpression of CBP partially rescued the neuronal loss observed in Drosophila, mouse and cellular models of the disease [112, 113, 115], whereas the genetic depletion of CBP further reduced the life span of HD mice bearing a hemizygous CBP mutation [135].

#### 2.2.2 Other poly-Q Disorders

Other poly-Q diseases share molecular mechanisms with HD and have also been linked to impaired KAT activity. This is the case for spinal and bulbar muscular atrophy (SBMA), which is caused by long poly-Q stretches in the androgen receptor, and spinocerebellar ataxia type 3 (SCA3), which is caused by mutations of the ataxin-3 gene. In experimental models of both diseases, CBP has been found sequestered in the protein aggregates [136], although the finding on SCA3 was not confirmed in a later study [122]. Interestingly, other KAT activities have been linked to different forms of spinocerebellar ataxia. Spinocerebellar ataxia type 7 is caused by mutations in the gene ataxin-7, which encode a component of the KAT GCN5-containing coactivator complexes [137]. *In vitro* experiments indicate that although mutant ataxin-7 does not disrupt such complexes, it interferes with their KAT activity [138, 139]. The scenario *in vivo* is more complex because KAT activity is apparently not affected in mouse models of the disease, but these mice exhibit a general chromatin de-condensation and aberrant histone acetylation in transcriptionally altered genes [140]. 

#### 2.2.3 Alzheimer’s disease 

Alzheimer’s disease (AD) is a progressive neurodegenerative disorder that causes the most common form of dementia in elderly people. Its most distinctive hallmark is the appearance of intracellular neurofibrillary tangles and extracellular amyloid plaques in cortex, hippocampus and amygdala [141]. Like in HD, several lines of evidence link KAT3 proteins and AD. First, prior to appreciable neurodegeneration, CBP is downregulated in the absence of presenilin 1 (PS1) and 2 (PS2) concomitantly with a reduction in the expression of CREB-dependent target genes and memory impairment [142]. These findings are consistent with the observation that PS1 variants containing mutations associated with familial AD (FAD) fail to positively regulate either CBP or p300 transcriptional activities [143, 144]. Moreover, CRE/CBP-dependent gene expression has been found to be altered in a high-throughput proteomic screen carried out in PS1 + AbetaPP mice [145]. Second and in apparent contradiction with the previous point, in culture experiments indicate that mutations in PS1 associated with FAD enhance CBP activity. Because the production of the N-Cad/CTF2 peptide is inhibited by these mutations and this peptide binds CBP and promotes its proteasomal degradation, N-Cad/CTF2 reduction causes an increase in CBP function and the upregulation of downstream targets like cFos [146]. Third, activation of amyloid precursor protein-dependent signaling reduces CBP levels in primary neuronal cultures [147], and Aβ peptide interferes with CREB signaling upstream of CBP [148, 149]. Fourth, neuronal histone acetylation has been found to be reduced in some mouse models of AD, and treatment with compounds that increase acetylation levels ameliorates or restores some deficits [150, 151]. Fifth, the nuclear traslocation of the p300/CBP inhibitor EP300-interacting inhibitor of differentiation 1 (EID1) is increased in the brains of AD patients, and the overexpression of EID1 results in reduced levels of histone H3 and p53 acetylation, and βIII-tubulin downregulation [152].

Interestingly, the KAT activity of PCAF has also been shown to be involved in AD. PCAF knockout mice develop a resistance to amyloid toxicity. This effect is mediated by PCAF regulation of the expression of proteins involved in Aβ generation and degradation. Therefore, modulating different KAT activities may offer a new route for the development of anti-amyloid therapies [36].

#### 2.2.4 Parkinson’s disease 

In contrast to most of the other disorders discussed here, Parkinson’s disease (PD), a degenerative disorder characterized by severe motor dysfunction and the death of dopamine-producing cells in the substantia nigra, has been linked to enhanced KAT activity. PD is triggered by environmental neurotoxicity and mutations or copy number variations in specific genes, such as α-synuclein (α-syn), whose misfolded version is a major constituent of Lewy bodies [153]. Native α-syn confers neuroprotection against neurotoxic insults. One proposed mechanism by which this beneficial effect is accomplished is the selective down-regulation of p300 levels (but not CBP levels) through a still unknown mechanism. This decrease in p300 could cause the hypoacetylation of the transcription factor NF-κB, thus interfering with the transcriptional activation of the proapoptotic PKCδ gene [154]. Indirectly supporting this model, another report found that exposure to the pesticide dieldrin, which is implicated in PD etiology, produces the opposite effect and induces both histone hyperacetylation and CBP accumulation in dopaminergic neurons [155].

#### 2.2.5 Other Neurodegenerative Conditions

CBP reduction, in parallel with histone hypoacetylation, is also observed in models of amyotrophic lateral sclerosis (ALS) [147], a fatal adult-onset neuromuscular disease characterized by the selective degeneration of upper and lower motor neurons, progressive muscle wasting and paralysis. In addition, research on a wide variety of neuropathological conditions, from brain ischemia to aging, indicates that histone deacetylation may be a common feature associated with neuronal malfunction and death in very different pathological contexts. This conclusion is based on the beneficial effects of treatment with inhibitors of HDAC activity but a direct link with KAT3 proteins is still missing. 

## 3 STRATEGIES FOR REINSTATEMENT OF KAT3 ACTIVITY 

The previous section discussed how different neuropathological conditions have been associated with dysregulation of histone acetylation and KAT3 activities. Most restoratives strategies aimed at correcting these conditions involve the use of drugs that inhibit HDAC proteins (referred as HDACis), but alternative approaches, such as the genetic or pharmacological enhancement of KAT3 activity could serve a similar aim, possibly with increased specificity [31]. Also, in some cases such as certain animal models of PD and FAD described above, KAT activity should be repressed rather than enhanced. In this section, we will briefly comment on the neuroprotective properties of HDACi drugs (see other articles in this special issue and recent reviews [156, 157] for a detailed review of these compounds) and discuss other therapeutic strategies that directly target KAT3 function (Fig. **3**).

### 3.1 HDACi

Diverse drugs inhibiting HDAC proteins are currently available. Their specificity is limited because they target several HDAC proteins [156]. Among the most used HDACis are sodium butyrate (NaB) and trichostatin A (TSA), which inhibit Class I and Class II HDACs, suberoylanilide hydroxamic acid (SAHA), which also inhibits HDACs from Class IV [8], and valproic acid (VPA), a Class I HDACs inhibitor that is commonly used as an anticonvulsant and mood-stabilizing agent. None of aforementioned HDACis inhibits Class III HDACs. A number of studies have shown that these compounds can potentiate different types of memory. NaB administration favors both the acquisition and the extinction of contextual fear conditioning [21, 26] and increases the duration of object recognition memory [158, 159]. Similar effects have been reported for other HDACis, such as TSA [67, 88, 160, 161], VPA [26], SAHA [8], and novel, more selective, compounds [37, 162, 163]. Interestingly, SAHA, NaB and TSA also facilitate the induction of long-term LTP (L-LTP) in the Schaffer collateral pathways [21, 54, 67], and chronic NaB treatment triggers remodeling of dendritic arbors and enhanced spine density [150].

Apart from their effects in wild type animals, many studies have reported beneficial effects of HDACi administration in animal models of different neuropathological conditions, from *Drosophila* and *C. elegans* to rodents. For example, HDAC inhibition prevents cell loss and increases cell survival in HD models [115, 117, 125, 164-166]. Although the most immediate effect is the restoration of the gene expression program [167-169], HDACis might also modulate CAG instability and enhance mHtt clearance [132-134]. Furthermore, HDACi administration recovers memory-related functions and enhances LTP in AD models [150, 151, 170-174]. Although there is little information about which HDAC isoforms are specifically targeted by these inhibitors, Class I HDACis seem to be the most effective in counteracting memory impairments in different AD models. HDACi treatment also ameliorates both motor performance deficits and the survival of motor neurons in an ALS mouse model, although there are some discrepancies between the existing studies (see review in [31]). Paradoxically, two HDACis, VPA and NaB, induce the expression of neuroprotective α-syn in PD models [175, 176], although it is not known whether this is the main mechanism involved in the observed amelioration of the symptoms. Histone deacetylation and reversal or attenuation of its associated deficits by HDACis have also been observed in animal models of other neuropathologies, like SBMA [177], ischemia [178-180], traumatic brain injury [181], intracerebral hemorrhage [182], and aging [183], to mention some prominent examples. These results may indicate either the existence of common pathways underlying all these conditions of very diverse origin or, more likely, the activation of neuroprotective mechanisms by HDACis that improve the chances of neuronal survival regardless of the identity of the insult. 

We should note that promoting indiscriminate acetylation can also have deleterious effects. Some experiments in cultured neurons have revealed neurotoxic effects associated with HDACi treatment [184-186] especially after chronic administration, whereas pulse treatments have neuroprotective properties [186]. Moreover, excessive NaB concentrations worsen the pathological traits during treatment of SBMA mice models, whereas lower concentrations produce their amelioration [177]. Interestingly, experiments in a cellular model of apoptotic neurodegeneration have demonstrated that the overexpression of either CBP or p300 significantly protects neurons in a pro-apoptotic condition (low K^+^) but is deleterious for neurons maintained in survival conditions [147]. The inverted U curve defining the relationship between histone acetylation and neuroprotection may explain some apparent contradictions between studies (Fig. **3**). For example, whereas a number of studies support a beneficial effect of HDACis in PD [175, 176], others indicate that HDACi administration can exacerbate the pathology [154, 184]. In conclusion, there exists a range of HDACi concentrations that is optimal for treatment, but if that range is exceeded, side effects may appear. Taken together, these results argue that the use of HDACis to treat neurodegenerative diseases will require fine-tuning and targeted delivery to the affected areas. 

### 3.2 KATe

Given the high level of interest in the development of compounds that enhance histone acetylation and the limited specificity and pleiotropic effects of the currently available HDACis, the effort to identify and design molecules that target KAT activation (i.e., a KAT enhancer or KATe) has run in parallel with the development of new, safer and more specific HDACis. However, very few molecules with such activity are known. The best characterized KATe is N-(4-Chloro-3-trifluoromethyl-phenyl)-2-ethoxy-6-pentadecyl-benzamide (CTPB), which was first found to enhance the KAT activity of p300 in cultured cells [187]. Unfortunately, CTPB was found to be cell-impermeable, which limits its therapeutic use. To address this problem, CTPB can be conjugated to carbon spheres, a strategy that has been used successfully to induce hyperacetylation of histone H3 in the mouse brain upon intra-peritoneal injections of the conjugate [188]. More recently, a second activator of p300 acetyltransferase suberoylanilide (a major constituent of floral resins) was identified in a screen of natural compounds. Its ability to penetrate cell membranes and modulate histone acetylation together with its high affinity for the KAT3 proteins in *in vitro* assays [189] have made this compound an attractive basis for the design of novel drugs aimed at restoring pathological histone deacetylation; however, the efficacy of such drugs *in vivo* still needs to be evaluated. Notably, targeting a different KAT has been more successful in enhancing memory formation. Thus, the infusion of the KAT2B/ PCAF activator pentadecylidenemalonate 1b (SPV106) enhances memory for fear extinction and prevents fear renewal [190], although we should be aware that this compound, like many HDACis, was initially described as a powerful pro-apoptotic drug in dividing cells [191].

### 3.3 Combined Strategies

HDACi and KATe drugs are aimed at restoring defective KAT activity. However, due to the highly modular structure of CBP/p300, it may be also desirable to restore other functions diminished by CBP and/or p300 deficiency, including their function as transcriptional co-activators. In this sense, phosphodiesterase 4 inhibitors (PDE4i), such as rolipram, prevent the hydrolysis of cAMP and increase PKA-dependent signaling upstream of CREB and CBP. Rolipram treatment of neuronal cultures and hippocampal slices reverses the effects of Aβ pathogenic peptide at the levels of CREB phosphorylation and LTP deficits [149] and dendritic spine alterations [186]. There is also evidence, including amelioration of learning impairments [186, 192], that this compound is beneficial in AD animal models. Interestingly, this type of inhibitor also improves learning and synaptic plasticity in wild-type animals of different ages [193, 194] and in RSTS models [54, 195]. Moreover, rolipram reduces CBP sequestration and neuronal loss and ameliorates motor impairments in a HD mouse model [196]. Based on this evidence, the combined use of both HDACi and PDE4i to synergistically increase their beneficial actions in the treatment of PS-based AD has been proposed [197]. This strategy may be extendable to other pathologies and to KATe. 

### 3.4 Genetic Approaches

Although its application is currently complicated, gene therapy represents a potential alternative to pharmacological approaches. Genetic approaches can be particularly powerful because, in contrast to HDACis that only address reduced KAT activity, multiple protein functions can be restored at once. As discussed in the previous sections, overexpression of KAT activity in transgenic animals has a beneficial effect in mouse and Drosophila models of HD. A similar approach based on a lentiviral vector, which thus has potential gene therapy applications, was recently tested in a mouse model of AD. That study found that viral delivery of CBP in the brain ameliorates learning and memory deficits. Notably, such improvements occurred without changes in Aβ and tau pathology and were linked to increased levels of brain-derived neurotrophic factor (BDNF) [198]. Based on these results, the authors proposed that increasing CBP expression in adult brains might be a valid therapeutic approach that may not be limited to AD but may also be effective in other brain disorders in which CREB-dependent gene expression is impaired. 

### 3.5 KATi

Therapies aimed at restoring normal KAT3 levels in situations in which KAT3 levels are pathologically enhanced, such as the experimental models of PD and FAD previously discussed, may be even more challenging than those addressing histone hypoacetylation. Few KAT inhibitors (KATi) are known, and it is likely that the indiscriminate or chronic reduction of histone acetylation would have deleterious effects. Among these KATis, we find two naturally occurring compounds: anacardic acid and curcumin that may have beneficial effects in pathological conditions like PD [155, 199, 200] and AD [201, 202]. The former compound inhibits CBP/p300, PCAF and Tip60, whereas the latter is more specific for CBP/p300 [203]. However, neither compound is very specific; in fact, curcumin also interferes with HDAC and DNA methyltransferase activities [204], which reduces its potential as an effective therapeutic drug. More specific KATis might result from the chemical modification of these natural compounds. Two currently available synthetic alternatives are Lys-CoA-Tat and C646, which selectively affect p300 and CBP [205-207]. Both have been reported to influence memory formation, although they do so in an unexpected manner. As with HDACis, pharmacological inhibition of CBP/p300 activity in the infralimbic prefrontal cortex (ILPFC) by C646 enhanced the extinction of fear memory and LTP in this area. These paradoxical observations cast the general role of KAT3 activities as positive regulators of memory formation in question and suggest that, at least in ILPFC, KAT3 proteins may constraint synaptic plasticity and memory [208].

## 4 PERSPECTIVES

The discovery of the therapeutic potential of activating histone acetylation in neurodegenerative disorders is relatively recent. Fortunately, a wide array of compounds with the potential to activate histone acetylation is already available and new compounds are under development largely thanks to the known efficacy of these compounds in treating different forms of cancer. However, the field still needs to overcome a number of conceptual and technical limitations. Basic questions regarding the function of HDACs and KATs still need to be clarified, and some controversies outlined in this review need to be solved. For instance, we still need to understand the specific roles of the different HDAC and KAT activities in neuronal function, identify the gene sets downstream of each of them, and clarify the requirement of normal histone acetylation levels for neuronal viability. From the technical point of view, prior to assessing the efficacy of HDACis, KATes and KATis in human patients, we need to thoroughly characterize their potential cytotoxicity and pleiotropic effects in animal and cellular models, decipher the gene expression profiles induced by the treatment refine their molecular specificity and improve their targeted delivery to affected brain regions. Many challenges lie ahead, but the prospects are positive and we are confident that new drugs for the treatment of neurological diseases will emerge from the current efforts. 

## Figures and Tables

**Table 1.** Neurological Traits in Mouse Strains with Deficient KAT3 Activity